# Comparison of LC-ESI, DART, and ASAP for the analysis of oligomers migration from biopolymer food packaging materials in food (simulants)

**DOI:** 10.1007/s00216-021-03755-0

**Published:** 2021-11-08

**Authors:** Jazmín Osorio, Margarita Aznar, Cristina Nerín, Christopher Elliott, Olivier Chevallier

**Affiliations:** 1 Analytical Chemistry Department, GUIA Group, I3A, EINA, University of Zaragoza, Mª de Luna 3, 50018 Zaragoza, Spain; 2 ASSET Technology Centre, Institute for Global Food Security, School of Biological Sciences, Queens University Belfast, 9, Belfast, Northern Ireland UK

**Keywords:** Oligomers, UPLC-Q-TOF–MS, DART, ASAP, Migration, Food contact material

## Abstract

Biopolymers based on polylactic acid (PLA) and starch have numerous advantages, such as coming from renewable sources or being compostable, though they can have deficiencies in mechanical properties, and for this reason, polyester resins are occasionally added to them in order to improve their properties. In this work, migration from a PLA sample and from another starch-based biopolymer to three different food simulants was studied. Attention was focused on the determination of oligomers. The analysis was first performed by ultraperformance liquid chromatography quadrupole-time-of-flight mass spectrometry (UPLC-Q-TOF–MS), which allowed the identification of the oligomers present in migration. Then, the samples were analyzed by two ambient desorption/ionization techniques directly coupled to mass spectrometry (ADI), direct analysis in real-time coupled to standardized voltage and pressure (DART-MS) and atmospheric pressure solids analysis probe (ASAP-MS). These methodologies were able to detect simultaneously the main oligomers migrants and their adducts in a very rapid and effective way. Nineteen different polyester oligomers, fourteen linear and five cyclic, composed of different combinations of adipic acid [AA], propylene glycol [PG], dipropylene glycol [DPG], 2,2-dibutyl-1,3-propanediol [DBPG], or isobutanol [i-BuOH] were detected in migration samples from PLA. In migration samples from starch-based biopolymer, fourteen oligomers from poly(butylene adipate co-terephthalate) polyester (PBAT) were identified, twelve cyclic and two linear. The results from ADI techniques showed that they are a very promising alternative tool to assess the safety and legal compliance of food packaging materials.

**Graphical abstract:**

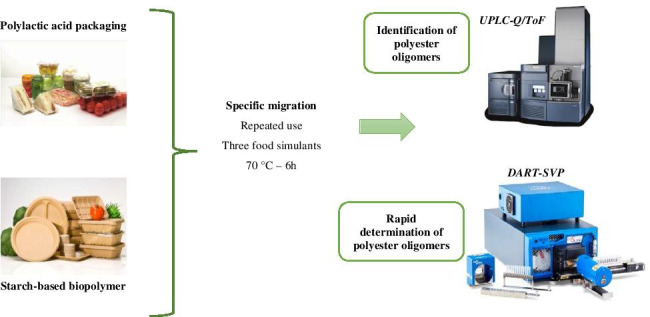

**Supplementary Information:**

The online version contains supplementary material available at 10.1007/s00216-021-03755-0.

## Introduction

More recently, the packaging industry is looking for more environmentally friendly materials that also have the mechanical characteristics of conventional packaging materials, such as its flexibility, strength, and thermal stability [1]. For this reason, the demand for biopolymers has increased over the last number of years, especially for polylactide (PLA) and starch-based polymers [2, 3]. This is because they are biodegradable and/or compostable under industrial conditions and come from renewable resources [4], making them suitable candidates to replace conventional plastics in the packaging sector [5]. For these materials, the addition of a biodegradable aliphatic–aromatic (co)polyester(BPES) is necessary, in most cases to improve their physicochemical properties [4]. For food contact materials (FCMs), polybutylene adipate terephthalate (PBAT) and polybutylene succinate terephthalate (PBST) are widely used as biodegradable polyesters [5].

Polyesters are manufactured by the polymerization of aliphatic diols, aliphatic dicarboxylic acids, and/or aromatic dicarboxylic acids during a polycondensation reaction [4, 6]. During the manufacturing process, oligomers can be also formed. These oligomers are considered nonintentionally added substances (NIAS) [7] and could potentially migrate from the FCM to the food, compromising consumers’ safety. Even though pure biopolymers are only regulated by the EU regulation 1935/2004/CEE, if they include some conventional materials or resins, the European Plastics Regulation, Regulation (EU) No 10/2011, should be applied. This legislation establishes a positive list with authorized substances in the manufacturing process, as additives and monomers, and their specific migration limits (SMLs) [7]. The maximum allowed concentration in migration for any substances not included in the positive list must be lower than 0.01 mg kg^−1^ food or food simulant [7]. The legislation establishes that the study of these substances should be done through migration studies with conditions similar to those of storage and food simulants that simulate alcoholic, acidic, and fatty foods.

In previous studies, some polyester oligomers from PLA and starch-based biopolymers were identified by UPLC-Q-TOF–MS [2]. The identification of other polyester oligomers is difficult because they are not included in any database. The use of hyphenated techniques, which combine chromatographic separation and high-resolution mass spectrometry, is very useful to achieve this purpose. Once the compounds are identified, other techniques such as ambient desorption/ionization (ADI) can be used in order to detect its presence in new samples. ADI techniques are commonly used for direct and rapid analysis of compounds present in solid or liquid samples [8, 9], since they allow a rapid confirmation of the presence of target compounds. They have been previously used in the study of different food packaging materials, for example, in the analysis of non-visible set-off components [10] and the quantitative determination of bisphenol A (BPA) [11].

In the present study, the two ADI techniques used were Direct Analysis in Real-Time (DART) and Atmospheric Pressure Solids Analysis Probe (ASAP). Both provided a direct sample analysis at ambient condition, a fast scan time and easy operation.

DART is one of the most popular ambient pressure ionization methods. In this technique, the sample is vaporized and afterwards, the molecules are ionized by excited helium molecules [12, 13]. Then, the ionized vapor is introduced into the detector for its analysis. In the ionization process, different adducts are commonly formed, such as [M + H]^+^ or [M + NH_4_]^+^ [9, 11]. The formation of adducts is promoted by the molecular weight, volatility, or polarity of the species present in the samples [14]. This technique has been successfully implemented for the detection of different analytes, such as the determination of BPA from thermal printing receipts and tickets [11], forensic screening [15], or food quality and safety control [16], among others. ASAP is also an ambient pressure ionization method for analyzing volatile or semi-volatile compounds (volatility below 500 °C) coming from liquids or solid materials [17]. This technique was successfully applied for the detection of nicotine and their metabolites [18] or for polyaromatic hydrocarbons [18]. Even though both techniques have a similar operating principle, in ASAP, the sample is introduced directly into the ionization chamber, improving the general sensitivity, except for the heaviest compounds where sensitivity decrease [17].

The aim of this work was to explore a direct method based on ADI techniques for the screening of polyester oligomers present in the migration samples from PLA and starch biopolymers used in food packaging. The structural elucidation of the linear or cyclic polyester oligomers detected was based on their parent ion exact mass and their fragmentation mass spectra. This analysis was performed by UPLC-Q-TOF–MS. Subsequently, DART-MS and ASAP-Q-TOF–MS techniques were used as tools to assess the presence of all polyester oligomers with a very short analysis time.

## Materials and methods

### Chemicals and reagents

Methanol (UHPLC-MS grade), ethanol absolute (HPLC grade), and acetic acid (HPLC grade) for the UPLC-Q-TOF–MS analysis and ASAP were supplied by Scharlab (Barcelona, Spain). Ethanol absolute (HPLC grade) for the analysis in DART was supplied by Merck (Darmstadt, Germany). Ultra-pure water was obtained from a Millipore Milli-Q system (Billerica, MA, USA).

### Samples

Biopolymers based on polylactic acid (PLA) and starch were supplied by a polymer manufacturing company for this study. Additional information about the sample cannot be provided. Samples were in the form of cups and dishes.

### Migration assays

The migration tests were established in accordance with the European legislation on food contact materials (Regulation No 10/2011/EU) [7]. Three simulants were evaluated: ethanol 10% (v/v) (simulant A), acetic acid 3% (w/v) (simulant B), and ethanol 95% (v/v) (simulant D2 substitute). Migration assays were carried out during 10 days at 70 °C. The assays were carried out by total immersion of the sample (5 cm × 2 cm) into 20 mL of the simulant.

### Analysis by UPLC-Q-TOF–MS

Chromatographic separation of the oligomers present in the migration solutions was performed using an Acquity UPLC from Waters Corporation (Milford, MA, USA) with a UPLC BEH C18 column of 1.7 μm particle size (2.1 × 100 mm). The chromatography parameters were 35 °C column temperature, 0.3 mL min^−1^ column flow, and 10 μL injection volume. The gradient elution was carried out with two mobile phases: (A) water with 0.1% formic acid and (B) methanol with 0.1% formic acid. The separation started at 98/2 (phase A/phase B), and at 8 min, it was changed to 0/100 (phase A/phase B) with two additional minutes at the final composition.

A quadrupole-time-of-flight mass spectrometer (Q-TOF–MS) Xevo G2 from Waters Corporation (Milford, MA, USA) with an ESI probe was coupled to the UPLC system. The following parameters were used: ESI + (positive ionization mode); sensitivity (analyser mode); 3.0 kV (capillary voltage); 30 V (sampling cone voltage); 3 V (extraction cone); 150 °C (source temperature); 20 L/h (cone gas flow rate); and 500 L/h (desolvation gas flow rate) at 450 °C (desolvation temperature). The acquisition was carried out in MS^E^ (acquisition mode), at low and high collision energy (CE) in the collision cell, in a mass range between m/z 50 and 1000.

### Analysis by DART-MS

Direct Analysis in Real-time Standardised Voltage and Pressure (DART) 201 model ion source (IonSense, Saugus, MA, USA) was operated with helium (grade A) in running mode and nitrogen in standby mode, with 3.5 L min^−1^ helium flow, temperature 150–450 °C, and ion-source grid voltage 350 V. The DART source was coupled to a Waters Acquity QDa Performance single quadrupole mass spectrometer (Waters Corporation, Manchester, UK), operated in positive ion mode via a Vapur interface (IonSense, Saugus, MA, USA), with desolvation line temperature 250 °C, source temperature at 150 °C, interface voltage at + 30 V and *m/z* 50–1000 scan range. Continuum data were acquired (scan time 0.5 s). The mass spectrometer was controlled using MassLynx v4.1 SCN888 (Waters Corporation, Wilmslow, UK). Data were analyzed by MassLynx v4.1. An aliquot of 3 μL of the migration solution was pipette-spotted directly onto the QuickStrip card. Then the QuickStrip card was then mounted on the sampling rail for analysis and passed orthogonally through the plasma source at a speed of 2 mm/s.

### Analysis by ASAP-Q-TOF–MS

The atmospheric pressure solids analysis probe (ASAP) was coupled to a quadrupole-time-of-flight mass spectrometer (Q-TOF–MS) Xevo G2 (Waters Corporation, Milford, MA, USA). The following mass spectrometer parameters were used: API + (positive ionization mode), source temperature at 120 °C, desolvation temperature at 450 °C, desolvation flow 650 L/h, and current corona at 5 µA. Three cone voltages were evaluated, namely, 30 V, 50 V, and 70 V, and 30 V was finally selected. The acquisition was carried out in the mass range between m/z 50 and 1000. Samples were directly introduced into the ASAP dipping previously a solid glass capillary in the migration samples. A blank, introducing the glass capillary in the migration blank was also performed. The analysis was acquired in SCAN continuous mode (scan time 0.5 s).

### Data processing

The UPLC-Q-TOF–MS and ASAP-Q-TOF–MS mass data were analyzed with MassLynx software V 4.1 from Waters (Milford, MA, USA). In both techniques, the mass spectra obtained in function 1 provided information about the elemental composition of the precursor ion and the mass spectra in function 2 provided information about the fragment ions. The identification methodology was optimized in previous works [2]. The DART mass spectra were acquired with MassLynx SCN888T software and processed with MassLynx software V 4.1.

## Results and discussion

### Identification of polyester oligomers by UPLC-Q-TOF–MS

The polyester oligomers found in migration samples from PLA and starch-based biopolymers are described in Tables 1 and 2, respectively. Since no commercial standards were available, identification was based on the structural elucidation of the peaks detected, which was performed thanks to the exact mass of the parent ion and the fragments obtained by UPLC-Q-TOF–MS analysis. The tables also show their retention time, their accurate mass, the adduct detected ([M + H]^+^ or [M + Na]^+^), their molecular formula, and the simulant in which they were detected. The chromatograms of migration samples can be seen in supplementary material, Figs. S1–S6.

**Table 1:** List of polyester oligomers detected in migrations samples from PLA by UPLC-Q-TOF–MS and DART-MS

UPLC-Q/ToF								Main adduct-DART-MS*				
*t*_R_	Mass	Adduct	MF	Candidate oligomer	Simulant			*m/z*	Adduct	%Ab	Adduct [M + NH_4_]^+^	%Ab
					A	B	D2					
5.79	187.1375	[M + H]^+^	C_9_H_14_O_4_	[AA-PG] *(cyclic)*	X	X	X	187.0	[M + H]^+^	4.7		
5.07	285.0988	[M + Na]^+^	C_12_H_22_O_6_	H-[AA-DPG]-OH (*linear)*	X	X	X	245.2	[M-H_2_O + H]^+^	4.0		
8.00	339.2166	[M + Na]^+^	C_17_H_32_O_5_	H-[AA-DBPG]-OH (*linear)*	X	X	X	299.3	[M-H_2_O + H]^+^	39.3		
6.65	395.1667	[M + Na]^+^	C_18_H_28_O_8_	2[AA-PG] *(cyclic)*	X	X	X	373.3	[M + H]^+^	10.6	390.3	1.6
6.04	413.1787	[M + Na]^+^	C_18_H_30_O_9_	H-2[AA-PG]-OH *(linear)*	X	X	X	373.3	[M-H_2_O + H]^+^	10.6		
6.29	471.2228	[M + Na]^+^	C_21_H_36_O_10_	H-[AA-DPG]-[AA-PG]-OH (*linear)*	X	X	X	431.4	[M-H_2_O + H]^+^	7.8		
8.27	525.3069	[M + Na]^+^	C_26_H_46_O_9_	H-[AA-DBPG]-[AA-PG]-OH (*linear)*	X	X	X	485.5	[M-H_2_O + H]^+^	60.5		
7.32	581.2668	[M + Na]^+^	C_27_H_42_O_12_	3[AA-PG] *(cyclic)*	X	X	X	559.4	[M + H]^+^	16.1	576.5	3.7
7.06	599.2734	[M + Na]^+^	C_27_H_44_O_13_	H-[AA-PG]_n_–OH *(linear)*	X	X	X	559.4	[M-H_2_O + H]^+^	16.1	594.4	3.5
8.44	653.3636	[M + Na]^+^	C_32_H_54_O_12_	[i-BuOH-AA- i-BuOH]-2[AA-PG] *(linear)*			X	631.5	[M + H]^+^	3.7	648.6	6.1
6.92	657.3203	[M + Na]^+^	C_30_H_50_O_14_	H-[AA-DPG]-2[AA-PG]-OH (*linear)*	X	X	X	617.5	[M-H_2_O + H]^+^	18.5		
8.40	711.5505	[M + Na]^+^	C_35_H_60_O_13_	H-[AA-DBPG]-2[AA-PG]-OH (*linear)*^**a**^		X	X	671.6	[M-H_2_O + H]^+^	100.0		
7.70	767.3526	[M + Na]^+^	C_36_H_56_O_16_	4[AA-PG] *(cyclic)*	X	X	X	745.7	[M + H]^+^	8.8	762.7	8.1
7.22	785.3586	[M + Na]^+^	C_36_H_58_O_17_	H-4[AA-PG]-OH *(linear)*	X	X	X	745.7	[M-H_2_O + H]^+^	8.8	780.7	1.2
8.60	839.4518	[M + Na]^+^	C_41_H_68_O_16_	[i-BuOH-AA- i-BuOH]-3[AA-PG] *(linear)*			X	817.8	[M + H]^+^	4.8	834.9	6.6
7.62	843.3873	[M + Na]^+^	C_39_H_64_O_18_	H-[AA-DPG]-3[AA-PG]-OH (*linear)*			X	803.7	[M-H_2_O + H]^+^	5.3		
8.49	897.4931	[M + Na]^+^	C_44_H_74_O_17_	H-[AA-DBPG]-3[AA-PG]-OH (*linear)*		X	X	857.8	[M-H_2_O + H]^+^	41.4		
7.98	953.4359	[M + Na]^+^	C_45_H_70_O_20_	5[AA-PG] *(cyclic)*			X	931.0	[M + H]^+^	14.1	948.1	26.2
7.63	971.4451	[M + Na]^+^	C_45_H_72_O_21_	H-5[AA-PG]-OH *(linear)*			X	931.0	[M-H_2_O + H]^+^	14.1		

**Fig. 1:**
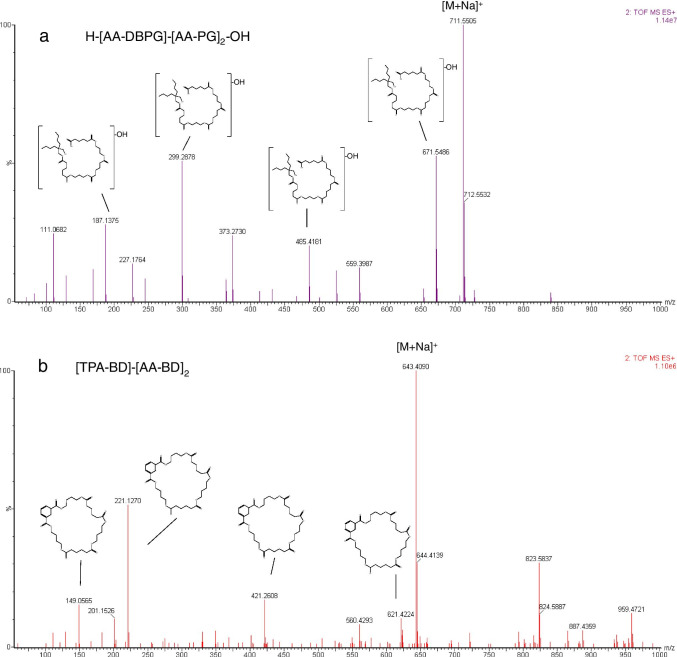
UPLC-Q-TOF–MS: High collision energy spectra for [AA-DBPG]-[AA-PG]_3_ oligomer from PLA (**a**) and [TPA-BD]-[AA-BD]_2_ polyester oligomer from starch-based biopolymer (**b**)

*t*_R_: retention time (min) in ethanol 95%. MF: molecular formula. Simulant A: ethanol 10% (v/v). Simulant B: acetic acid 3% (w/v). Simulant D2: ethanol 95% (v/v). Int: relative intensity. % Ab: % relative abundance. ^**a**^Oligomer from Fig. 1a. *Oligomers detected in specific migration samples to Simulant D2

**Table 2:** List of polyester oligomers detected in migrations samples from starch-based biopolymer by UPLC-Q-TOF–MS and DART-MS

UPLC-Q/ToF								Main adduct-DART-MS*			
*t*_R_	Mass	Adduct	MF	Candidate oligomer	Simulant			Adduct [M + H]^+^	%Ab	Adduct [M + NH_4_]^+^	%Ab
					A	B	D2				
7.50	221.1270	[M + H]^+^	C_12_H_12_O_4_	[TPA-BD] *(cyclic)*			X	221.1	5.0		
5.50	223.1412	[M + Na]^+^	C_10_H_16_O_4_	[AA-BD] *(cyclic)*			X	201.1	4.2	218.2	1.3
5.55	313.1660	[M + Na]^+^	C_14_H_26_O_6_	[AA-BD]-[BD] *(linear)*	X	X	X	291.2	11.8	308.3	3.9
7.00	423.1984	[M + Na]^+^	C_20_H_32_O_8_	2[AA-BD] *(cyclic)*	X	X	X	401.3	13.1	418.4	18.5
6.40	441.2093	[M + H]^+^	C_24_H_24_O_8_	2[TPA-BD] *(cyclic)*	X	X	X	441.3	1.6	458.4	3.3
7.50	443.1684	[M + Na]^+^	C_22_H_28_O_8_	[TPA-BD]-[AA-BD] *(cyclic)*	X	X	X	421.3	12.6	438.4	15.7
6.64	513.3770	[M + Na]^+^	C_24_H_42_O_10_	2[AA-BD]-[BD] *(linear)*	X	X	X	491.5	7.5	508.5	14.4
7.77	623.3054	[M + Na]^+^	C_30_H_48_O_12_	3[AA-BD] *(cyclic)*	X	X	X	601.5	27.6	618.5	58.2
8.09	643.4090	[M + Na]^+^	C_32_H_44_O_12_	[TPA-BD]-2[AA-BD] *(cyclic)* ^**a**^			X	621.4	50.0	638.5	100.0
8.34	663.3835	[M + Na]^+^	C_34_H_40_O_12_	2[TPA-BD]-[AA-BD] *(cyclic*			X	641.4	24.3	658.5	61.4
8.75	683.3540	[M + Na]^+^	C_36_H_36_O_12_	3[TPA-BD] *(cyclic)*			X	661.5	5.5	678.5	16.3
7.98	823.4092	[M + Na]^+^	C_40_H_64_O_16_	4[AA-BD] *(cyclic)*			X	801.8	7.7	818.8	12.5
8.23	843.5593	[M + Na]^+^	C_42_H_60_O_16_	[TPA-BD]-3[AA-BD] *(cyclic)*			X	821.7	16.3	838.7	28.5
8.48	863.3457	[M + Na]^+^	C_44_H_56_O_16_	2[TPA-BD]-2[AA-BD] *(cyclic)*			X	841.7	8.6	858.7	18.0

*t*_R_: retention time (min) in ethanol 95%. MF: molecular formula. Simulant A: ethanol 10% (v/v). Simulant B: acetic acid 3% (w/v). Simulant D2: ethanol 95% (v/v). Int: relative intensity. %Ab: % relative abundance

^**a**^Oligomer from Fig. 1b. *Oligomers detected in specific migration samples to Simulant D2

The analysis of PLA-based migration sample revealed that a polyester resin was used during the manufacturing of the biopolymer. It was composed by one kind of polyacid, adipic acid [AA]; three different kinds of polyols, namely, propylene glycol [PG], dipropylene glycol [DPG], and 2,2-dibutyl-1,3-propanediol [DBPG]; and one alcohol, isobutanol [*i*-BuOH]. Polyacids and polyols are commonly used during the manufacturing of polyesters [19, 20], and its presence in the final material will help to the elucidation of the polyester used. Table 1 shows the presence of nineteen different polyester oligomers in the migration samples from PLA, where fourteen of them were cyclic and five were linear. The main monomers found were C_9_H_14_O_4_ [AA-PG], C_12_H_21_O_5_ [AA-DPG], C_17_H_31_O_4_ [AA-DBPG], and C_14_H_27_O_4_ [-i-BuOH-AA-i-BuOH]. Their respective dimers, trimers, tetramers, or different combinations among them were also observed. Simulant D2 was the simulant with the highest number of oligomers (nineteen oligomers), followed by simulant B (fourteen oligomers) and simulant A (twelve oligomers). Therefore, these compounds had a higher tendency to migrate to fat food.

In this analysis, five series of oligomers were found. The first series corresponds to cyclic oligomers with the structure [AA-PG]_n_ (*n* = 1 to 5). The second series is similar to the first one but with the addition of a water molecule and opening the ring H-[AA-PG]_n_–OH (*n* = 2 to 5), resulting in a linear oligomer. Other series of linear oligomers found were H-[AA-DPG]-[AA-PG]_n_–OH (*n* = 0 to 3), H-[AA-DBPG]-[AA-PG]_n_–OH (*n* = 0 to 3), and finally, [i-BuOH-AA- i-BuOH]-[AA-PG]_n_ (*n* = 2 to 3).

Table 2 shows the oligomers found in migration from the starch-based polymer. A total of fourteen oligomers composed by butanediol [BD] and two different kinds of diacids, terephthalic acid [TPA] or adipic acid [AA], were detected. Some of these oligomers were previously reported by different authors as coming from poly(butylene adipate co-terephthalate) polyester (PBAT) [2]. Twelve were cyclic oligomers and two were linear oligomers. The main monomers found were C_10_H_17_O_4_ [AA-BD] and C_12_H_13_O_4_ [TPA-BD]. All of them were found in simulant D2, since in simulants A and B, the same six oligomers were observed. Their respective dimers, trimers, tetramers, or different combinations among them were also observed. Four series of cyclic oligomers with the following structures were detected: [TPA-BD]_n_ (*n* = 1 to 3); [AA-BD]_n_ (*n* = 1 to 4); and [TPA-BD]_m_-[AA-BD]_n_ (*m*/*n* = 1 to 3). In addition, a series of linear oligomers was also observed: [AA-BD]_n_-[BD] (*n* = 1 to 2).

Figure 1a shows the high collision energy spectra of one of the main oligomers detected in migration from PLA-based material, H-[AA-DBPG]-[AA-PG]_2_-OH (8.40_711.5505). Two of the fragment ions detected have been previously reported in the literature as part of a common fragmentation spectra of a polyester used in a PLA-based biopolymer [21]. The fragment ions corresponded to m/z 187.1375 and 111.0682, which were associated to the formulas C_9_H_15_O_4_ ([AA-PG]_1_) and C_6_H_7_O_2_ (monomer [AA]), respectively.

Figure 1b shows the high collision energy spectra of one of the main cyclic oligomers detected in migration from starch-based biopolymer, [TPA-BD]_1_-[AA-BD]_2_ (8.09_643.4090), coming from PBAT polyester. In this case, common fragment ions were observed at m/z 149.0565 that corresponded to the monomer formula [TPA] (C_8_H_4_O_3_) [22].

### DART analysis of polyester oligomers in migration samples

**Fig. 2:**
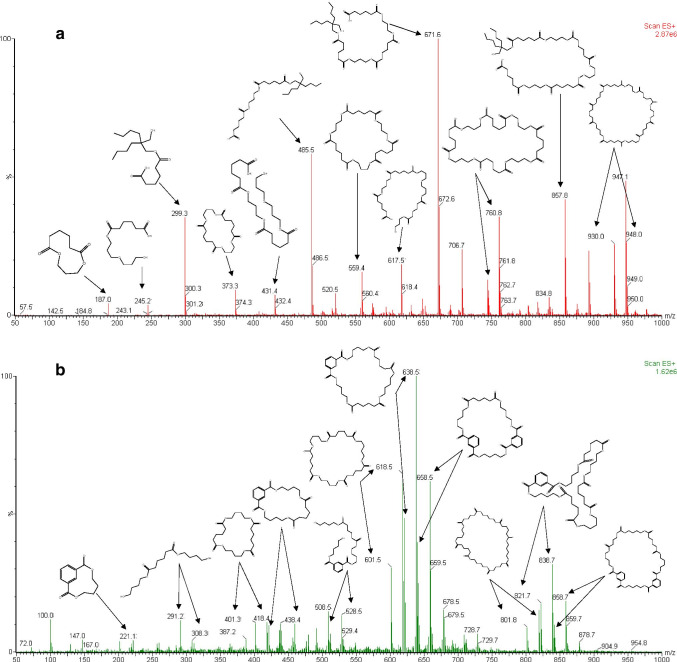
DART-MS spectrum of migration assay in EtOH 95% from PLA biopolymer (**a**) and starch-based biopolymer (**b**). The adducts for each of the polyester oligomers are linked to Tables 1 and 2

The ability to implement ADI techniques as a rapid methodology to determine the polyester oligomers coming from biopolymers was investigated. Table 1 shows the main adducts of the polyester oligomers detected in PLA migration samples by DART, as well as their m/z and relative abundance. All the oligomers were previously detected and identified in migration samples by UPLC-Q-TOF–MS. Figure 2a shows a DART mass spectrum of a simulant D2 migration sample from the PLA sample. The structure and adducts of the candidate oligomers can be also observed in that figure.

The highest abundance in the mass spectrum was observed for m/z 671.6 (100%), corresponding to the adduct [M-H_2_O + H]^+^ of the linear oligomer with structure H-[AA-DBPG]-[AA-PG]_2_-OH. The following ions with high abundance corresponded to m/z 485.5 (60.5%), 857.8 (41.4%), and 299.3 (39.3%).

Similarly, Table 2 shows the main adducts of PBAT oligomers ([M + H]^+^ and [M + NH_4_]^+^) detected in migration samples of starch-based biopolymer by DART, and its relative abundance. In addition, six adducts were only detected by DART in the same migration samples of simulant D2 and their m/z values and the candidates proposed are shown in Table 3. The structure and molecular formula of the six candidates were calculated on the basis of the monomer units used in the manufacturing of PBAT polyester. Combining the monomers [TPA], [AA], and [BD] and the knowledge obtained from previous work carried out in our research group [22], six candidates with polyester oligomer structures were proposed. This strategy was previously used by E. Brandly [20] to identify other oligomers. Figure 2b shows a DART mass spectrum and the structure of the candidate oligomers present in the starch-based biopolymer sample. The highest abundance in the mass spectrum was observed for m/z 638.5 (100%), corresponding to the adduct [M + NH_4_]^+^ of the cyclic oligomer with structure [TPA-BD]-[AA-BD]_2_. The ions with the following high abundances corresponded to m/z 658.5 (61.4%), 618.5 (58.2%), and 621.4 (50.0%).

**Table 3:** Ions detected only by DART-MS in specific migration samples of ethanol 95% (v/v) from starch-based biopolymer

Candidate	MF	Main adduct			Other adducts
		*m/z*	adduct	%Ab	
[TPA-BD]-[BD] *(linear)*	C_16_H_22_O_6_	311.2	[M + H]^+^	4.0	
[TPA-BD]-[AA-BD]-[BD] *(linear)*	C_26_H_38_O_10_	528.5	[M + NH_4_]^+^	12.9	[M + H]^+^ (5.4)
2[TPA-BD]-[BD] *(linear)*	C_28_H_34_O_10_	531.4	[M + H]^+^	3.6	[M + NH_4_]^+^ (3.0)
3[AA-BD]-[BD] *(linear)*	C_34_H_58_O_14_	691.6	[M + H]^+^	4.3	[M + NH_4_]^+^ (3.1)
[TPA-BD]-2[AA-BD]-[BD] *(linear)*	C_36_H_54_O_14_	728.7	[M + NH_4_]^+^	6.4	[M + H]^+^ (4.1)
3[TPA-BD]-[AA-BD] *(cyclic)*	C_46_H_52_O_16_	878.7	[M + NH_4_]^+^	3.3	[M + H]^+^ (1.6)

MF: Molecular formula. Int: relative intensity. % Ab: % relative abundance

In DART, high polarity compounds generate adducts such as [M + H]^+^, [M-H + H_2_O]^+^, [M-H + O]^+^, or [M + NH_4_]^+^; and medium polarity compounds form adducts as [M]^+^, [M + H]^+^, or [M-H + O]^+^ [14, 23]. In PLA and in starch-based samples, the adducts [M + H]^+^, [M-H + H_2_O]^+^ and [M + NH_4_]^+^, [M + H]^+^, were observed, respectively. Detection of these adducts is very common in the analysis of oligomers in different polymers by the DART technique [24]. The identified adducts were carried out considering the possible interaction between the molecular ions of polyester oligomers and the species detected in the environment (oxygen, water, and ammonia).

The adducts that showed the highest abundance in PLA and in starch-based samples were H-[AA-DBPG]-[AA-PG]_n_–OH (*n* = 1 to 4) and the [TPA-BD]_m_-[AA-BD]_n_ (*m*/*n* = 1 to 3), respectively. Their high abundance could be attributed to a high concentration of these compounds in migration samples (Figures S1–S6). It has to be also taken into account that adducts detected in DART could come from oligomers belonging to oligomer series with similar structures, and therefore, common fragments (Fig. 1).

**Fig. 3:**
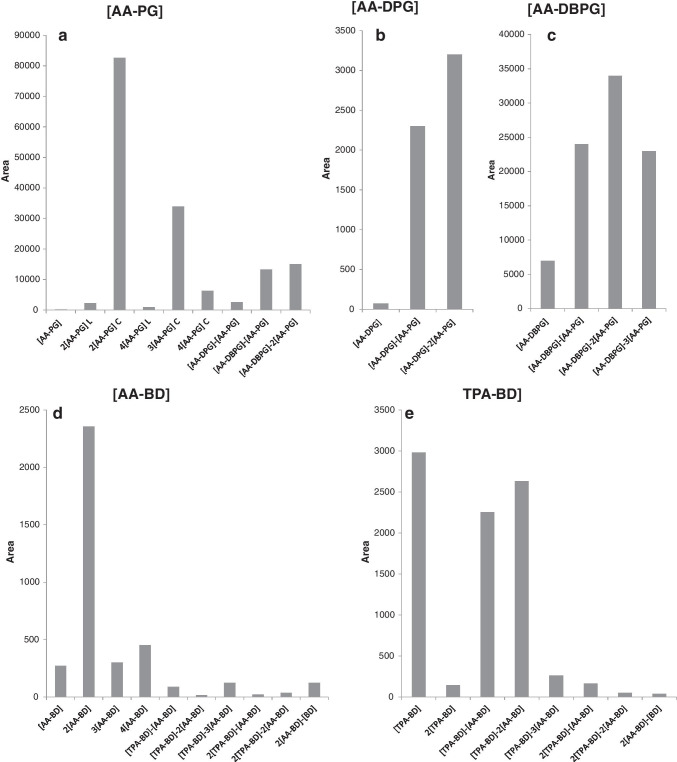
Area of the peaks obtained in the UPLC-Q-TOF–MS chromatograms of migration assay in EtOH 95% from PLA biopolymer (**a**–**c**) and starch-based biopolymer (**d**, **e**) when m/z corresponding to different monomers were extracted: [AA-PG] (187.2130), [AA-DPG] (245.2921), [AA-DBPG] (299.4256), [AA-BD] (201.2396), and [TPA-BD] (221.2292)

This fact can be specially observed for those DART m/z values corresponding to the monomers, such as [AA-PG] (187.0), [AA-DPG] (245.2), and [AA-DBPG] (299.3). When the m/z values corresponding to these structures were extracted in the UPLC-Q-TOF–MS chromatogram, it was observed that they were also present in heavier oligomers. Figure 3a–c shows the areas of the peaks detected in the chromatogram when the exact m/z values corresponding to these monomers were extracted. Therefore, the presence of m/z 187.0, 245.2, and 299.3 in a DART spectrum will inform the analysts about the presence of oligomers containing the monomers [AA-PG], [AA-DPG], and [AA-DBPG], respectively. With this information, the formula of different possible combinations within them to form oligomers can be calculated and its presence in the DART spectrum can be checked.

Figure 3d–e shows the areas of the peaks detected in the chromatogram when the exact m/z values corresponding to monomers [AA-BD] (201.1) and [TPA-BD] (221.1) were extracted. As it was previously described, these monomers are related to the use of a PBAT polyester. Therefore, the presence of m/z 201.1 and 221.1 in the DART spectrum would indicate that the sample could contain PBAT in its structure. As it was described for polyesters present in PLA-based polymers, the formula of the different monomers combination can be calculated and its presence can be checked in the DART spectrum to confirm its presence.

### ASAP-Q-TOF–MS analysis of polyester oligomers in migration samples

**Fig. 4:**
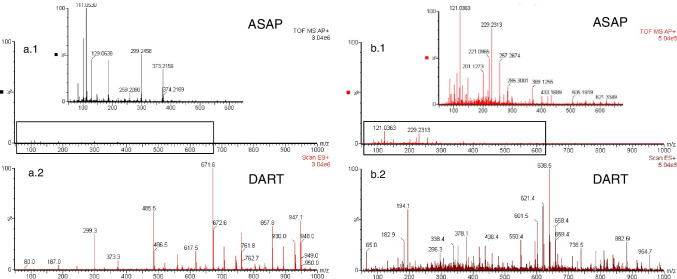
Comparison between DART-MS spectrum (**a.1**) and ASAP-Q-TOF–MS spectrum (**a.2**) of PLA migration sample in ethanol 95%. DARTDART spectrum (**b.1**) and ASAP-Q-TOF–MS spectrum (**b.2**) of starch-based biopolymer migration sample in ethanol 95%

The mass spectra in Fig. 4 correspond to a migration sample of PLA analyzed by ASAP-Q-TOF–MS (a.1) and DART (a.2), and a migration sample of a starch-based biopolymer analyzed by ASAP-Q-TOF–MS (b.1) and DART (b.2).

In the ASAP spectrum of PLA, Fig. 4a.1, the ion with the highest abundance corresponded to m/z 111.0530 that was linked to [AA] monomer. Also, the m/z values corresponding to oligomers [AA-PG] (m/z 187.0716), H-[AA-DBPG]-[AA-PG]-OH (m/z 299.2458), and [AA-PG]_2_ or H-[AA-PG]_2_-OH (m/z 373.2156) could be observed. Furthermore, these m/z values were detected in the DART mass spectrum of the same sample (Fig. 4a.2) but at lower abundances.

In the ASAP mass spectrum of starch-based biopolymer in Fig. 4b.1, m/z 121.0363 and 229.2376 had the highest relative abundance in the spectrum. These and other characteristic m/z of the PBAT oligomers were not detected in DART. Unidentified ions could correspond to volatile and semi-volatile compounds present in the sample [3]. They were not identified because it was not the objective of this work. On the other hand, m/z 201.1273, 221.0965, 421.2215, and 621.3349 corresponded to the oligomers [AA-BD], [TPA-BD], [TPA-BD]-[AA-BD], and [TPA-BD]-[AA-BD]_2_, respectively, previously detected in DART analysis.

Protonation and charge transfer are very common ionization mechanisms in ASAP, but the mechanism mainly depends on the polarity of the analyte [18, 25]. Polar molecules have a high affinity for protons; therefore, polyester oligomers (polar molecules) tend to form protonated adducts in ASAP [25]. In both samples, the ions of the detected oligomers corresponded to their [M + H]^+^ adducts. Unlike DART technique, [M + NH_4_]^+^ adducts are not common in ASAP.

Finally, Fig. 4 showed that, for both biopolymers, the adducts coming from the polyester oligomers had a higher abundance in DART than those in ASAP mass spectra. On the other hand, small ions showed a higher abundance in ASAP analysis than in DART analysis and therefore, this technique would be suitable for the analysis of smaller molecules. Therefore, the DART method has better sensitivity determination of polyester oligomer high molecular weight in migration samples from biopolymers, but ASAP could be a good alternative to analyze volatile or semi-volatile oligomers with volatility below 500 °C [18].

It can be pointed out that ASAP spectrum provides higher abundances of the lowest ions, such as 111.0530 or 129.0638, that would confirm the presence of AA in the monomer [19].

## Conclusion

Although biopolymers such as starch or PLA intended for food contact are considered ecological alternatives to conventional polymers, they are commonly blended with polyester resins to improve their mechanical properties so they could not be considered as pure biopolymers. Several polyester oligomers were found in migration samples from PLA and starch-based biopolymers, showing that polyester resins have a critical role in the evaluation of the material. This work showed that direct MS analysis techniques, such as DART and ASAP-Q-TOF–MS, are powerful tools for rapid and simultaneous determination of polyester oligomer present in migration from biopolymers samples. By DART analysis, it was possible to detect those polyester oligomers in a mass range between m/z 50 and 1000 and in a unique analysis of 1.5 min duration for each replicate. In the case of ASAP-Q-TOF–MS, only those polyester oligomers with small molecular mass were observed, and hence, this technique will be mainly applied to the screening of volatiles and semi-volatile polyester oligomers. The use of DART and ASAP would allow performing a quick detection of the presence of oligomers coming from polyesters. If the characteristic ions were detected, additional target analyses by UPLC-MS, focused on oligomers identification and quantification, would be needed. Nevertheless, if they were not detected, the additional analysis would not be necessary.

## Supplementary Information

Below is the link to the electronic supplementary material.

Supplementary file1 (PDF 920 kb)
