# Supplementary material for: Comparison of LC-ESI, DART, and ASAP for the analysis of oligomers migration from biopolymer food packaging materials in food (simulants)
Source: Anal Bioanal Chem. 2021 Nov 8;414(3):1335–45. doi: 10.1007/s00216-021-03755-0 (PMC8724096; doi:10.1007/s00216-021-03755-0)

SUPPLEMENTARY MATERIAL. Chromatograms of migration samples from biopolymer food packaging materials (PLA and starch based biopolymers)

Comparison of LC-ESI, DART and ASAP for the analysisof oligomers migration from biopolymer food packaging materials in food(simulants)

Jazmín Osorio[a,b], Margarita Aznar[a*], Cristina Nerín[a], Christopher Elliott[b], Olivier Chevallier[b]

[a] Analytical Chemistry Department, GUIA Group, I3A, EINA, University of Zaragoza, Mª de Luna 3, 50018, Zaragoza, Spain.

[b] ASSET Technology Centre, Institute for Global Food Security, School of Biological Sciences, Queens University Belfast, 9 Northern Ireland, UK.

***Corresponding Author**: Margarita Aznar Ramos

E-mail: marga@unizar.es

Figure S1. UPLC-Q-TOF-MS chromatograms of PLA biopolymer migration (food simulant: Ethanol 10%)

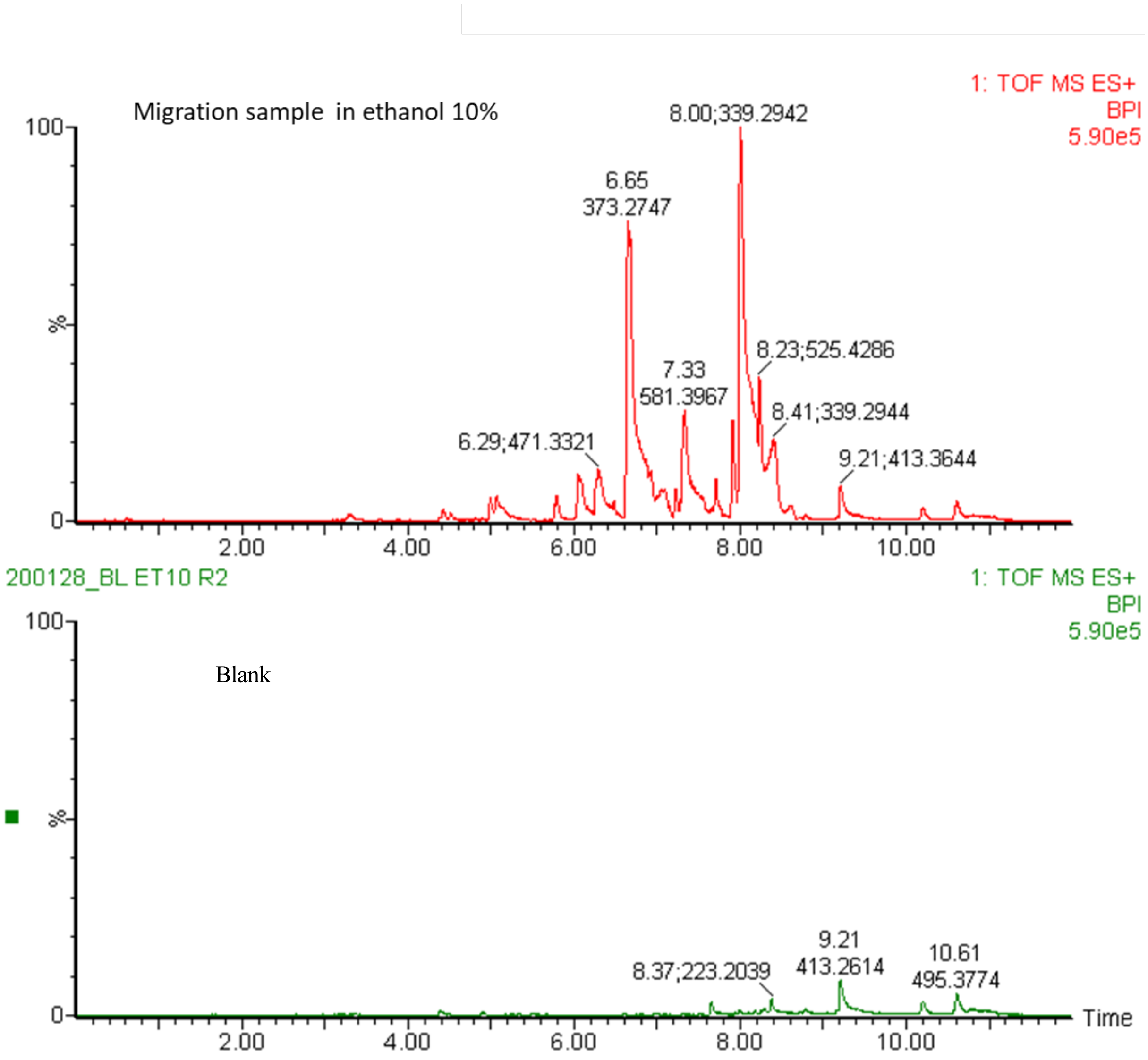

Figure S2. UPLC-Q-TOF-MS chromatograms of PLA biopolymer migration (food simulant: Ethanol 95%)

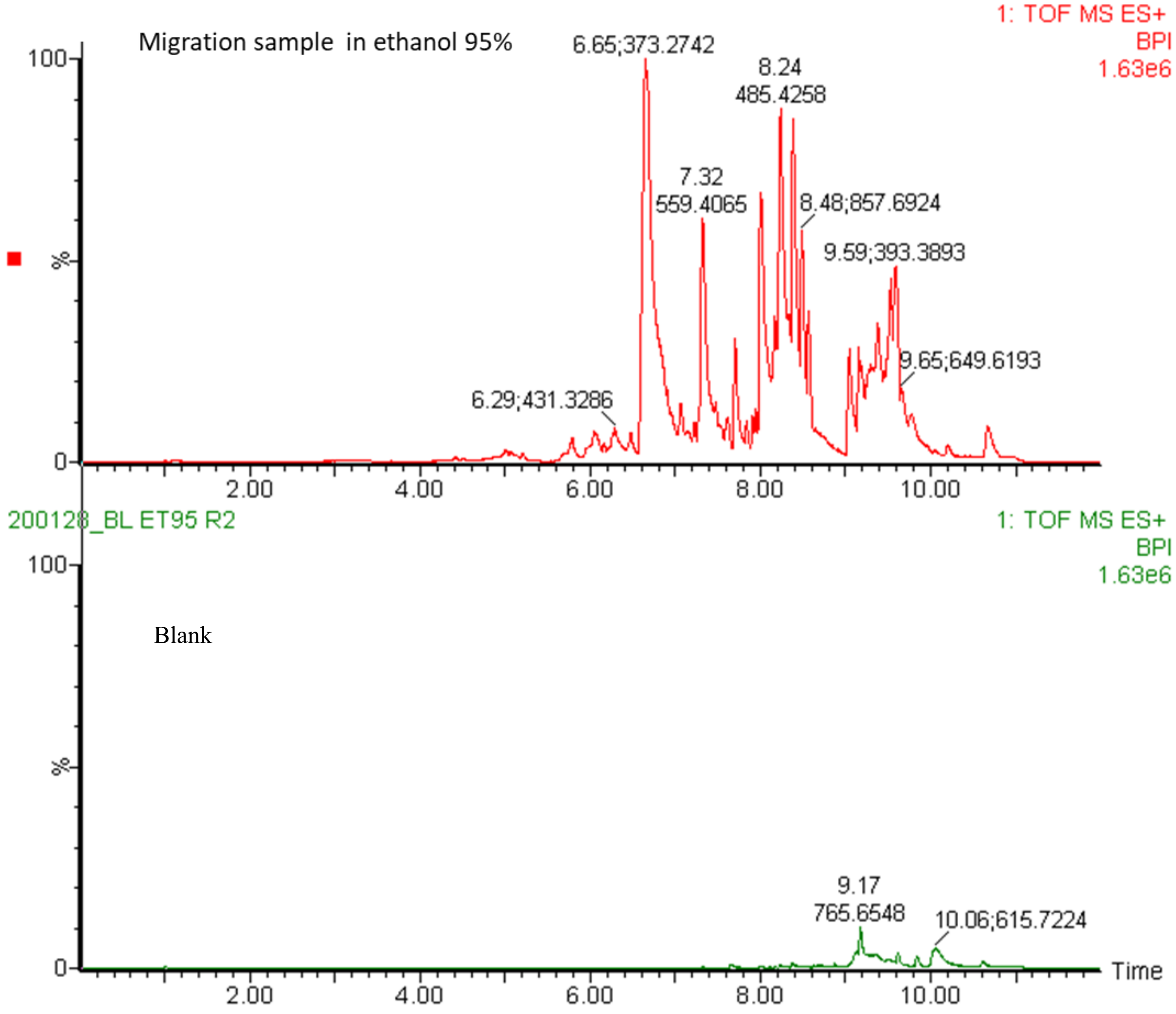

Figure S3. UPLC-Q-TOF-MS chromatograms of PLA biopolymer migration (food simulant: acetic acid 3%)

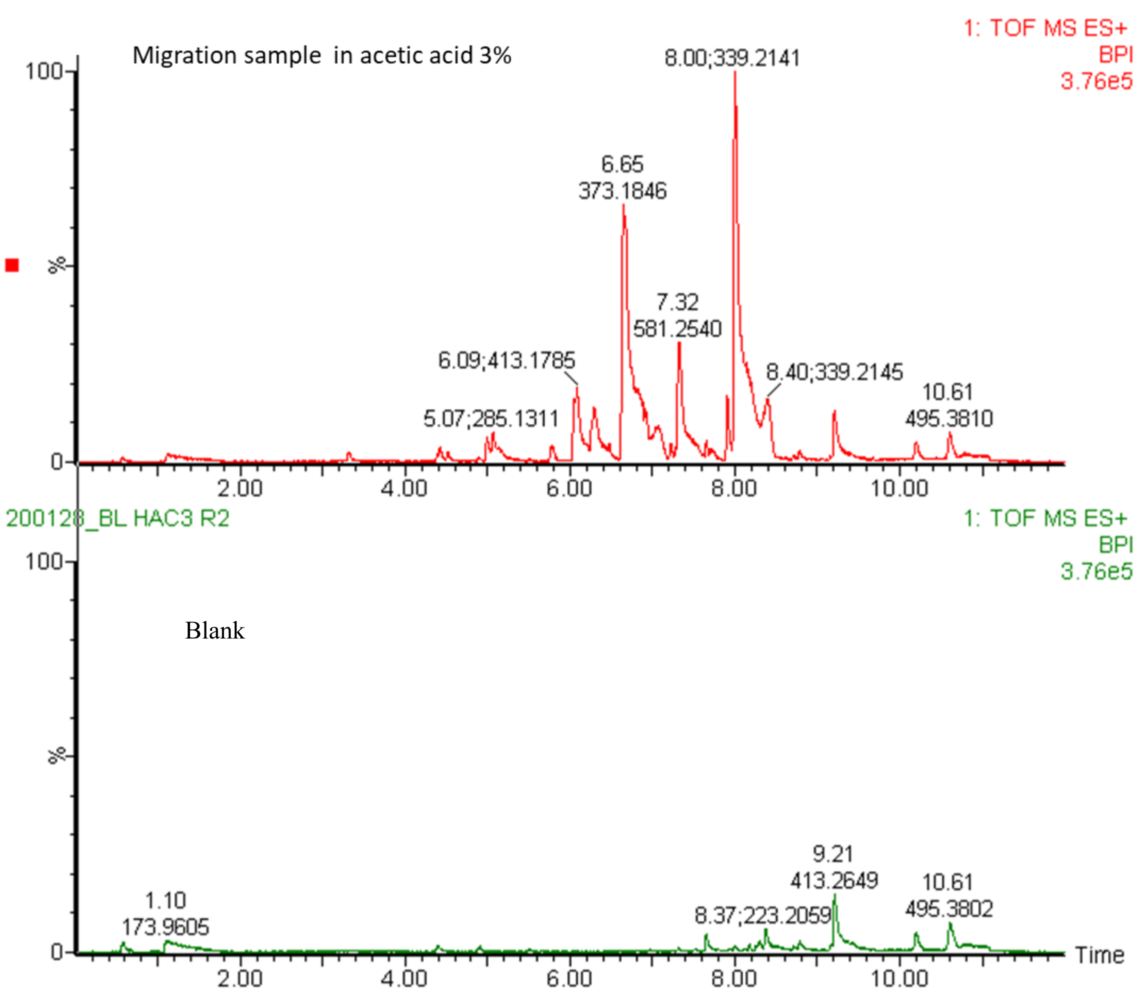

Figure S4. UPLC-Q-TOF-MS chromatograms of starch-based biopolymer migration (food simulant: Ethanol 10%)

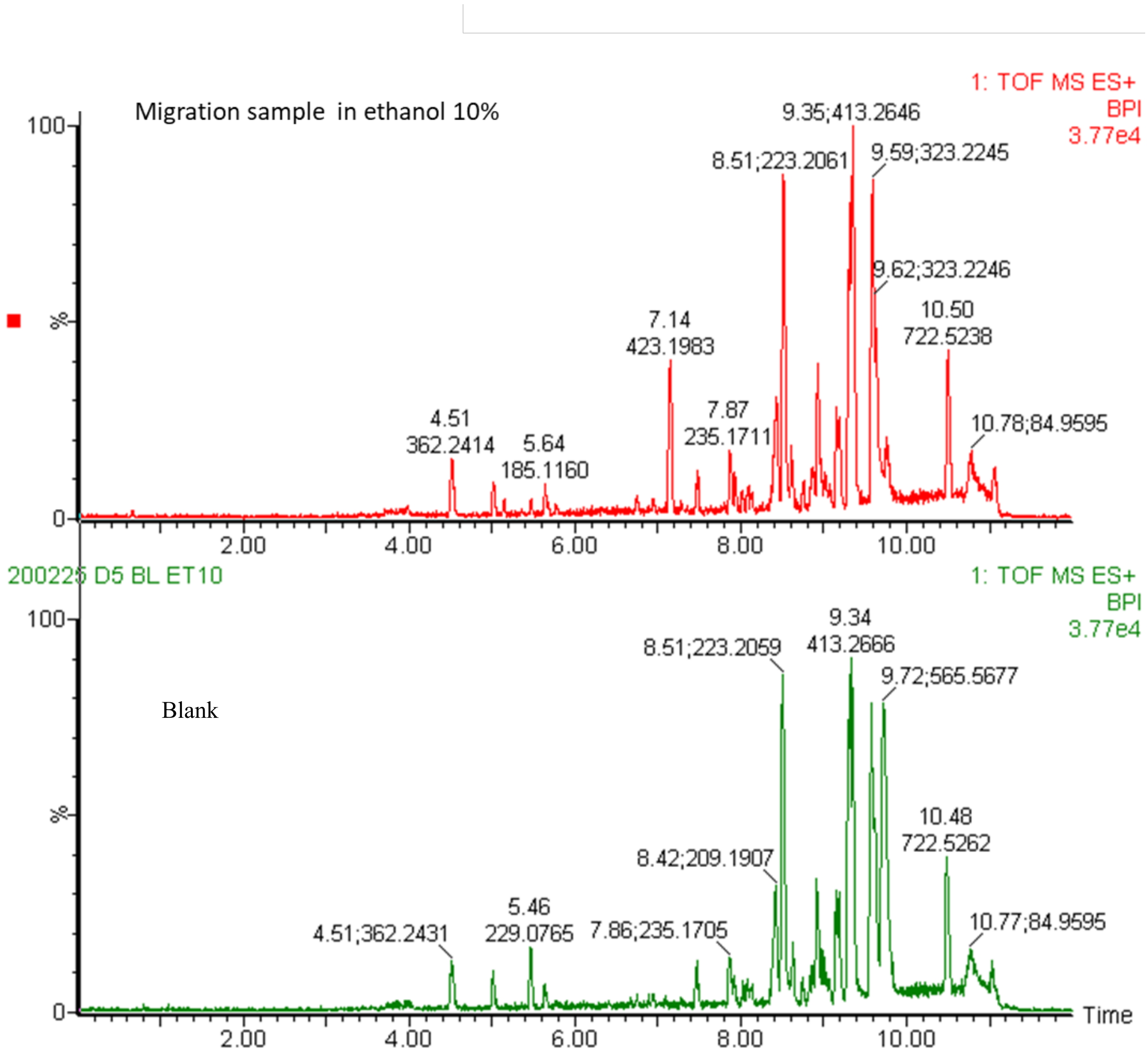

Figure S5. UPLC-Q-TOF-MS chromatograms of starch-based biopolymer migration (food simulant: acetic acid 3%)

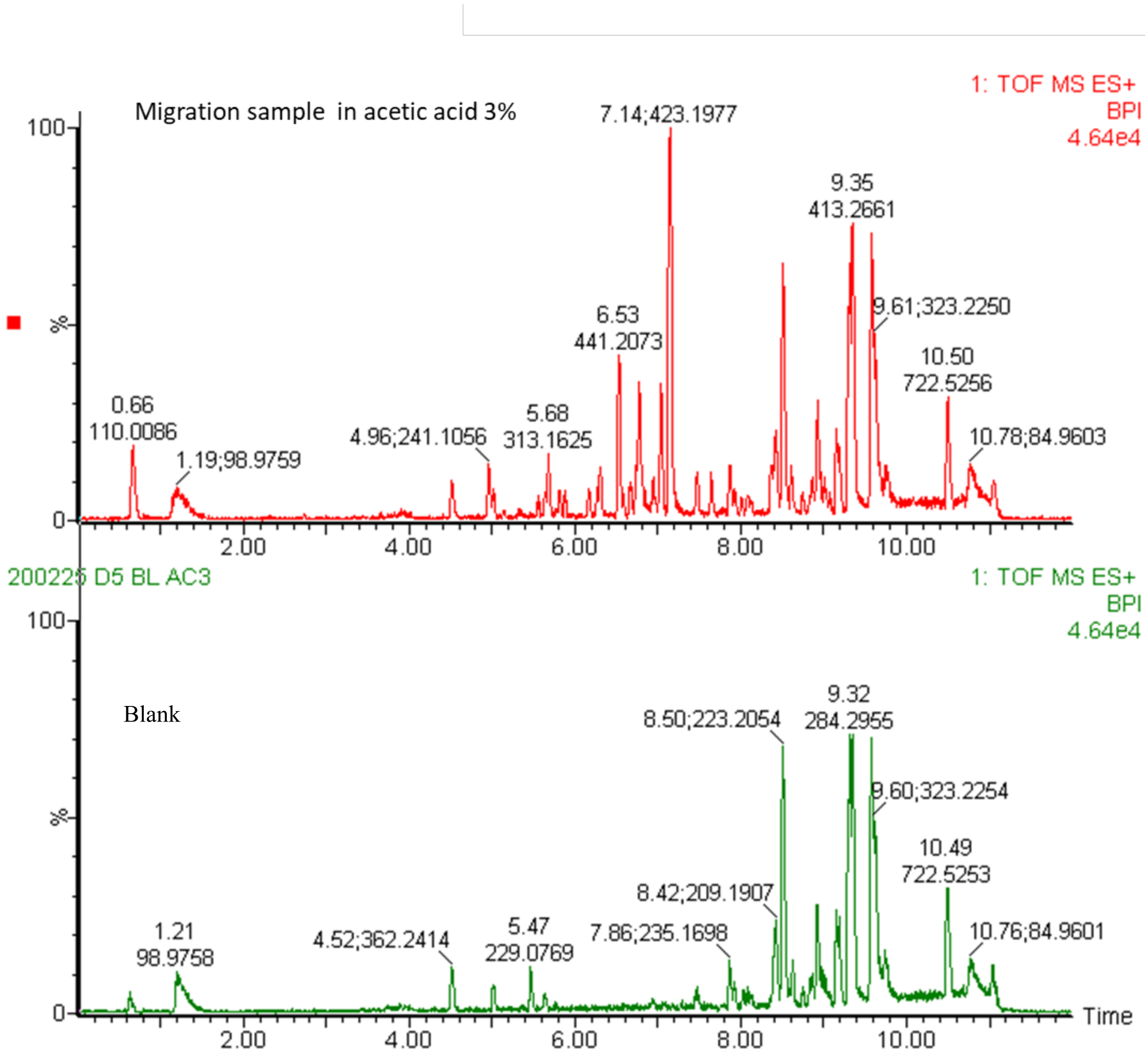

Figure S6. UPLC-Q-TOF-MS chromatograms of starch-based biopolymer migration (food simulant: Ethanol 95%)

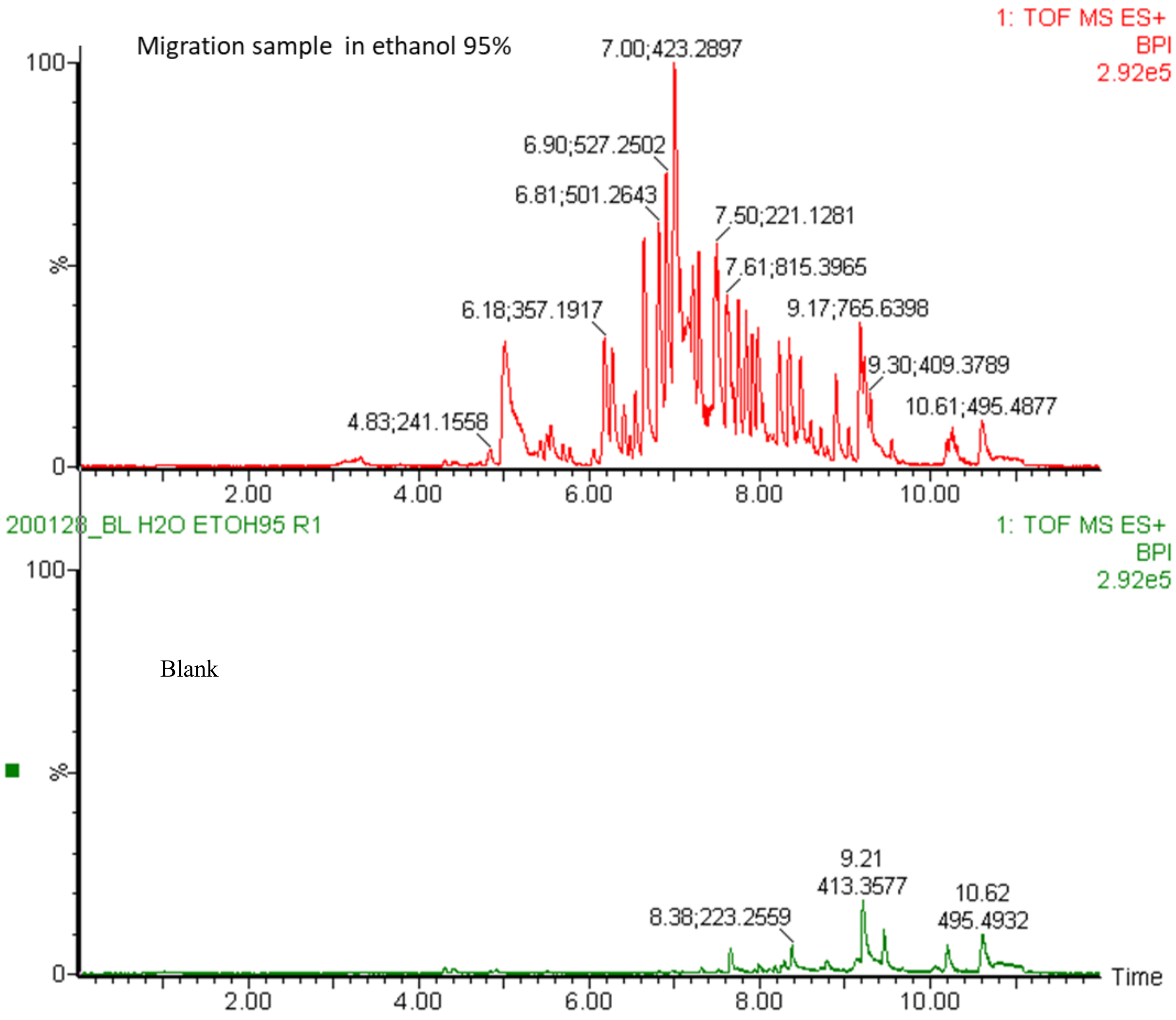

Supplement: Supplementary file 1 — Supplementary file1 (PDF 920 kb) [file 216_2021_3755_MOESM1_ESM.pdf]
